# A Language Model–Powered Simulated Patient With Automated Feedback for History Taking: Prospective Study

**DOI:** 10.2196/59213

**Published:** 2024-08-16

**Authors:** Friederike Holderried, Christian Stegemann-Philipps, Anne Herrmann-Werner, Teresa Festl-Wietek, Martin Holderried, Carsten Eickhoff, Moritz Mahling

**Affiliations:** 1 Tübingen Institute for Medical Education (TIME) Medical Faculty, University of Tübingen Tübingen Germany; 2 Department of Medical Development, Process and Quality Management, University Hospital Tübingen Tübingen Germany; 3 Institute for Applied Medical Informatics, University of Tübingen Tübingen Germany

**Keywords:** virtual patients communication, communication skills, technology enhanced education, TEL, medical education, ChatGPT, GPT: LLM, LLMs, NLP, natural language processing, machine learning, artificial intelligence, language model, language models, communication, relationship, relationships, chatbot, chatbots, conversational agent, conversational agents, history, histories, simulated, student, students, interaction, interactions

## Abstract

**Background:**

Although history taking is fundamental for diagnosing medical conditions, teaching and providing feedback on the skill can be challenging due to resource constraints. Virtual simulated patients and web-based chatbots have thus emerged as educational tools, with recent advancements in artificial intelligence (AI) such as large language models (LLMs) enhancing their realism and potential to provide feedback.

**Objective:**

In our study, we aimed to evaluate the effectiveness of a Generative Pretrained Transformer (GPT) 4 model to provide structured feedback on medical students’ performance in history taking with a simulated patient.

**Methods:**

We conducted a prospective study involving medical students performing history taking with a GPT-powered chatbot. To that end, we designed a chatbot to simulate patients’ responses and provide immediate feedback on the comprehensiveness of the students’ history taking. Students’ interactions with the chatbot were analyzed, and feedback from the chatbot was compared with feedback from a human rater. We measured interrater reliability and performed a descriptive analysis to assess the quality of feedback.

**Results:**

Most of the study’s participants were in their third year of medical school. A total of 1894 question-answer pairs from 106 conversations were included in our analysis. GPT-4’s role-play and responses were medically plausible in more than 99% of cases. Interrater reliability between GPT-4 and the human rater showed “almost perfect” agreement (Cohen κ=0.832). Less agreement (κ<0.6) detected for 8 out of 45 feedback categories highlighted topics about which the model’s assessments were overly specific or diverged from human judgement.

**Conclusions:**

The GPT model was effective in providing structured feedback on history-taking dialogs provided by medical students. Although we unraveled some limitations regarding the specificity of feedback for certain feedback categories, the overall high agreement with human raters suggests that LLMs can be a valuable tool for medical education. Our findings, thus, advocate the careful integration of AI-driven feedback mechanisms in medical training and highlight important aspects when LLMs are used in that context.

## Introduction

For most medical problems, history taking is the cornerstone of the diagnostic journey. Despite the increase in diagnostic tools such as advanced imaging and molecular and laboratory assays, a comprehensive history is necessary to guide further steps and may sometimes even be sufficient for diagnosing a disease without further testing [1,2]. Conversely, insufficient history taking can risk patients’ safety [3,4]. Due to its importance, history taking is taught to health care students worldwide, usually as part of a communication-focused curriculum or clinical clerkship [5-8] and mostly relying on real patients [9].

To enable more student-patient interactions without increasing costs, staff’s workload, or the burden on patients, virtual simulated patients have emerged as an adjunctive approach [10,11]. For communication skills in particular, web-based chatbots have been developed to offer an additional learning format [12], and recent advances in artificial intelligence (AI) such as large language models (LLMs) have helped those tools to achieve a new level of realism [13-15]. Indeed, recent work has demonstrated that OpenAI’s Generative Pretrained Transformer (GPT) model is capable of providing realistic, positively perceived patient experiences as well as scenarios requiring the breaking of bad news, all of which are simulated [13,16].

However, patient experiences alone are hardly sufficient to develop competence. Indeed, no matter the amount of their exposure to patients, medical students have to have feedback in order to progress in their performance [17,18]. Traditional teaching methods require teachers’ significant involvement in providing feedback, either while history taking is performed or in assessing the results afterward. LLM-based education, by contrast, offers the opportunity for repeated, unsupervised exposure to simulated patients. Whereas traditional virtual patients often yield low levels of feedback [10], the linguistic capabilities of LLMs can provide students with higher-quality feedback [19]. LLMs have also demonstrated the capability of providing feedback in other circumstances, including argumentation [20], writing [21], and scientific papers [22]. However, their capability to provide feedback on the quality of history taking has not been elucidated on a large scale, and concerns about the accuracy of AI-based feedback persist [23].

Building on our previous work showing that GPT-3.5 can provide simulated patient experiences [13], we evaluated the extension of our chatbot with an integrated feedback system while using the latest LLM model, GPT-4. In particular, we aimed to investigate whether GPT-4 can provide structured feedback on medical students’ performance during history-taking dialogs with a simulated patient, with special focus on such feedback’s realism and educational use. We hypothesized that GPT-4, given its capabilities in medical knowledge [24-26] and reasoning [13], can accurately assess students’ performance in history taking despite potential limitations such as logical errors [27] and AI’s propensity to generate nonsensical content, known as “hallucinations” [28]. Our objective was to evaluate feedback on medical students’ history taking provided by GPT and compare it with human feedback, all to contribute to the broader discourse on integrating AI into medical education.

Considering all of the above, we formulated the following research questions for our study:

1. What are the characteristics of medical students’ history-taking conversations (ie, question length and chain questions) with a GPT-4–powered simulated patient chatbot?
2. What is the quality of the GPT-4–powered chatbot’s role-play during such conversations (ie, are the questions answered and are the answers medically plausible)?
3. How is the history-taking dialog rated by GPT-4 and a human rater in terms of feedback topics covered?
4. How does GPT-4’s feedback compare with the feedback of a human rater (ie, interrater reliability)?
5. How can significantly different feedback between GPT-4 and the human rater regarding certain topics be explained?

## Methods

### Study Outline

We conducted a prospective study in which students performed a written history-taking exercise with a GPT-powered simulated patient (for more information, see [13]). Afterward, GPT-4 was prompted to provide the students feedback on the topics covered in the history taking. The chat history was analyzed in detail, and the GPT model’s feedback was compared with feedback from a human rater.

### Setting and Participants

During a scheduled break in a skills training course involving multiple opportunities for practice, medical students were asked to participate at an additional training station affording the opportunity to participate in history taking with our GPT-powered chatbot. Participation was voluntary. Given our study’s exploratory nature and aim to broadly assess the use of GPT-powered feedback in medical education, we did not impose any specific inclusion or exclusion criteria on participation beyond the willingness and ability to engage with the chatbot. Neither of those components was associated with any examination outcomes.

**Figure 1:**
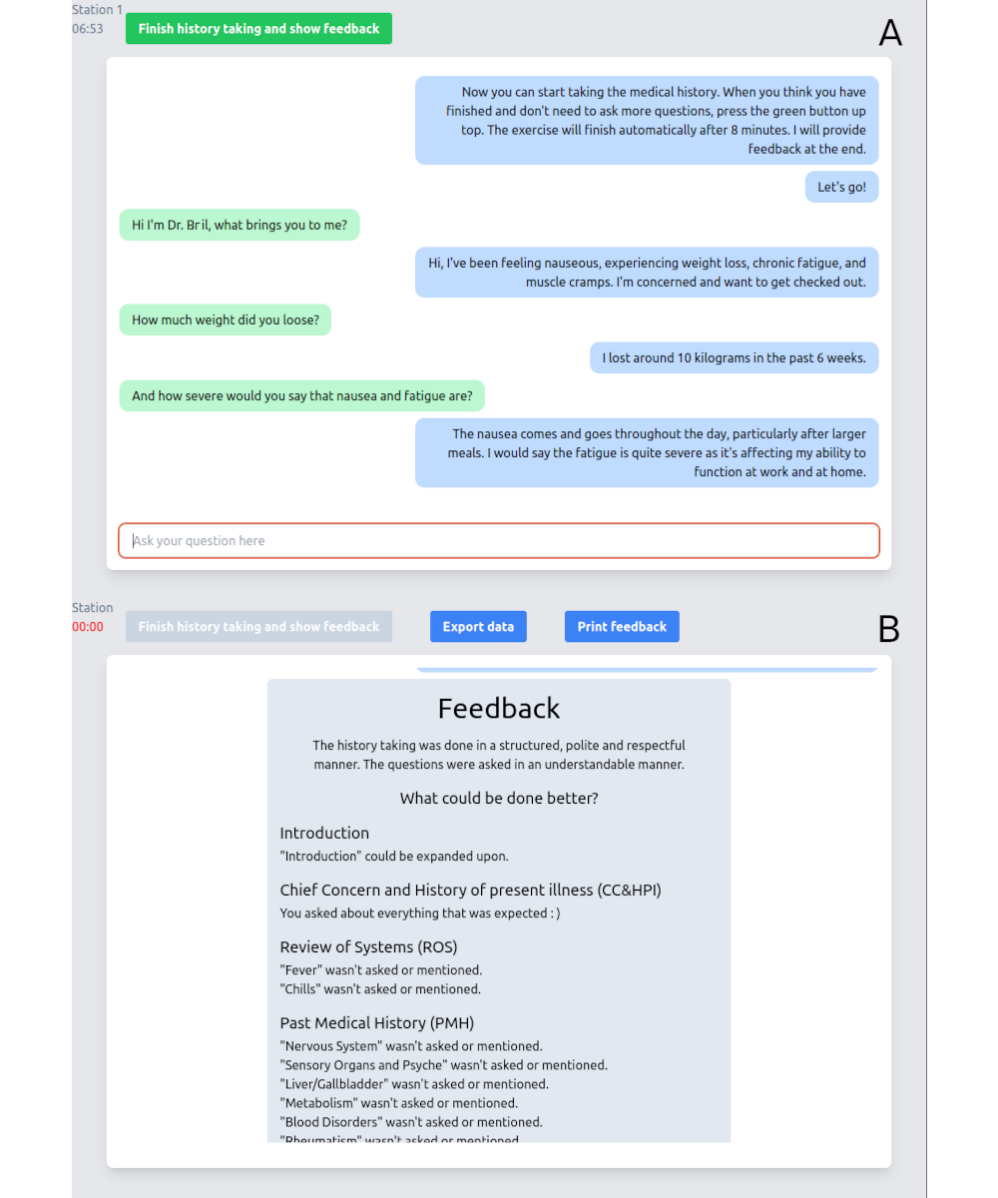
Screenshot of the chatbot interface as presented to participants (translated from German): (A) the interface during the interactive dialog and (B) the interface presenting the feedback.

The training station consisted of a laptop with the chat interface already prepared (Figure 1A). Given the course in which our station was embedded, the time limit for history taking was set to reflect the time limit of other stations (ie, 8 minutes). After finishing history taking, students were presented with AI-generated feedback (Figure 1B) and proceeded to the next practice station.

Our chat platform was a major update to the platform for history taking previously detailed by our group [13]. In short, we embedded GPT-4, accessed via an application programming interface (API), in a web page in order to enable participants to ask questions to a virtual simulated patient. Model parameters were left at their default settings, and the full chat history was anonymized and saved for further analysis.

### Prompt Development

Two prompts were developed: one for providing the interactive history-taking dialog, and the other for giving feedback.

### Behavioral Prompt

For the interactive history taking, we used an updated version of the prompt previously developed by our group [13]. In brief, we provided the model with a script describing an illness (“illness script”) and used an additional behavioral prompt to make the model behave as a virtual simulated patient. For the updated version of the behavioral prompt used in our study, the prompts for history taking were mostly upgraded by adding sentences describing intended or unintended behavior. We made those upgrades because the earlier prompts made the model too verbose or willing to provide assistance only in certain cases. We added more specific instructions, including that the model should generally answer in 1 sentence or 2, never ask a question unless specifically asked to do so, and never offer assistance.

Moreover, we provided tailored examples of how the simulated patient should respond to certain inputs—for instance, to respond with “OK” if no question was asked. Such modifications aimed to correct for intrusive model behavior in which the model sometimes provided its own question in response to a participant simply writing an affirmation or “OK.”

### Feedback Prompt

To make the GPT model generate feedback, we used an entirely different prompt. By calling the API, it is possible to gain full control of any message history that the model can access, as opposed to the common web interfaces of chatbots. In our case, that meant that the prompt for history taking and the prompt for feedback could not influence one another unless we intentionally reused parts of one in the other.

We used the illness script, as described in [13] and already used in the prompt for history taking, to define the categories by which to provide feedback, called “feedback categories.” Next, for each category of the illness script, the model’s task was to judge whether the information had appeared in the chat between the user and the simulated patient or whether it had been asked about. The main dilemma was, thus, the existence of 2 primary sources of information—the illness script and the chat—which complicated what the model paid attention to.

Our strategy was to begin with a description of the task, namely that the model needs to check whether the dialog that follows contains certain information and needs to answer a few questions at the end. We then provided categories from the illness script as fully phrased requirements in the form of “There should have been mention of X in the dialogue, with possible mention of Y”, in which case “X” was a category and “Y” the information given in the illness script. We used that strategy to guide the attention of the model before providing the chat. An example of such a construct was “In the dialogue, ‘Previous illnesses related to the main symptom’ should have been discussed, including information such as ‘I’ve never been like this before. I was usually healthy before.’”

We next pasted the complete chat, scaffolded with “=== START DIALOG ===” and “=== END DIALOG ===” to indicate that the content was a single long block quotation. As previously described [13], we inserted additional formatting into the chat to be presented in the prompt for history taking. However, those modifications were unnecessary and thus absent in the chat reproduced in the feedback prompt—that is, the chat was reproduced in the same way it would be shown to participants.

Following the dialog part, we again described the task of checking the dialog for certain information. We subsequently told the model that we would repeat the feedback categories and information from the illness script in a highly compact format, which we also added to the prompt. Last, we formulated the main question—“Did these categories appear in the dialog?”—and asked the model to give its answer in the form of a JSON dictionary, a computer-readable, structured way of representing key-value pairs and special feature available in recent GPT models [29]. Using the JSON dictionary allowed us to parse the answer of the model in our interface in order to compute scores for participants.

Another problem was that the amount of information in the prompt was liable to led to exceptionally long prompts. We also observed that inquiring about all categories simultaneously led to a high probability of scrambled answers, in which categories were not fully reproduced in the answers or were simply wrong. Despite the plausibility of asking about 1 category of the illness script at a time and issuing different API calls for each, sometimes called the “divide-and-conquer” strategy [30], doing so in our case may have easily overloaded the limits set by OpenAI for model usage or led to very high computing cost. We, therefore, decided to ask about a certain number of categories at a time and issue prompts for each of those small lists. In small initial experiments, limiting the number of categories to 8 tended to provide a good balance between accuracy and cost.

The full prompt is available as Multimedia Appendix 1.

### Analysis of the Characteristics of Conversations

Descriptive methods were used to characterize the conversations and question-answer pairs (QAPs), in which each question was inputted by participants and the answer was outputted by GPT. First, we calculated basic metrics to describe those QAPs, including the number of QAPs per conversation and the number of words per question and answer. To analyze the students’ questions in detail, they were scored for the presence of the following undesirable behavior when asking for a medical history:

1. Foreign terms (eg, use of medical terminology);
2. Chain questions (ie, asking more than one question);
3. Single-word questions (eg, typing simply “smoking” as a question); and
4. Incomprehensive wording or barely understandable input (eg, nonconclusive series of words or spelling mistakes).

### Analysis of the Quality of GPT-4’s Role-Play

To assess whether the quality of the GPT’s role-play was suitable for a simulated patient experience, as we have shown and analyzed in detail using GPT-3.5 [13], we had the answers outputted by GPT in the first 20% of the conversations assessed by rater FH and discussed with rater MM in equivocal cases. We assessed whether the participant’s questions were covered by our illness script, whether each answer matched the question, and whether the answer was medically plausible. That process was guided by categories and definitions developed in previous research [13]. Table 1 shows the categories, possible scores, and examples from the conversations.

**Table 1:** Areas, categories, and examples for the dialogs for the rating process, adapted from [13].

Areas, categories, and examples			Dialogues
**Student’s question covered by the illness script**			
	Question not covered by the script	Student: “Or was someone in your surroundings ill?” GPT: “No, as far as I know, no one in my immediate surroundings was ill. Both my sons are currently fit, and my wife hasn’t been feeling sick either.”	
	Question partly or indirectly covered by the script	Student: “And you mentioned feeling listless as well. Are you also experiencing a loss of interest and a low mood?” GPT: “I primarily feel listless, and it’s difficult for me to attend to my family duties after a long day at work. But I wouldn’t say that I’m experiencing a loss of interest or a low mood.”	
	Question explicitly covered by the script	Student: “And do you have any other symptoms like fever or night sweats?” GPT: “No, no fever. But I do occasionally experience episodes of increased sweating, though not only at night and not very intensely.”	
	Statement (no question)	Student: “Thank you and see you soon!” GPT: “See you soon!”	
**GPT’s response matches the question**			
	GPT’s response matches the question	Student: “Do you have a loss of appetite?” GPT: “Yes, my appetite is indeed much worse than before. Maybe also because I feel frequently more nauseous.”	
	GPT’s response does not match the question	No example occurred	
	Not applicable	For QAPs with answers provided for a previous question	
**Answer medically plausible (regarding case and illness script)**			
	+1: Answer plausible, relevant to case	Student: “What was your starting weight?” GPT: “I previously weighed 115 kg. Now I weigh only 105 kg.”	
	0: Answer plausible, not case-specific	Student: “All right!” GPT: “Okay.”	
	–1: Answer implausible	No example occurred	
	Not applicable	For QAPs with missing answers due to technical reasons	

### Analysis of the Completeness of History Taking

The completeness of the medical history for the prespecified topics was assessed by GPT-4 (see “Feedback Prompt”) and by a human rater (FH). To extract the feedback from GPT-4, we used the JSON file. For the human feedback, the rater assessed each QAP for the categories covered in a Microsoft Excel (version 16.0.10394.20022) spreadsheet. Both data sets were imported into R (version 4.3.1; The R Foundation) [31] for statistical analysis and figure generation. We calculated Cohen κ to compare the feedback from GPT-4 and the human rater on the chat using the R function “CohenKappa” from the “DescTools” package. Categories with κ < 0.6 were further examined by raters FH and MM in order to identify possible explanations.

All numerical data were assessed for normal distribution and, in this article, are presented as means and standard deviations. If the data deviated from a Gaussian distribution, then we provided the median and interquartile range (Q25-Q75).

### Ethical Considerations

This study was approved by the Ethics Committees of the Faculty of Medicine at Tübingen University Hospital (605/2023BO2). Participation in the study was voluntary, without any compensation, and data was collected anonymized. All methods were implemented in accordance with the Declaration of Helsinki.

## Results

### Participants’ Demographic Data

Of the 111 students asked to participate, 5 could not due to experiencing technical problems with the interview platform. All remaining 106 students agreed to participate; 78 (73.6%) identified as female, 25 (23.6%) as male, and 3 (2.8%) as nonbinary, and participants were 22.8 (SD 3.7) years old on average. As for progress in medical school, 93% of participants (N=99) were in their third year of medical school, whereas the remaining participants were in their first (2/106, 2%), second (1/106, 1%), or fourth (3/106, 3%) years, and one student provided an implausible answer (1/106, 1%). No student had to be excluded from the analysis.

### Characteristics of Conversations

In a total of 106 conversations, 1920 QAPs were recorded. Of them, 26 QAPs (1.4%) had to be excluded due to a missing server response, which left 1894 QAPs for analysis. Each conversation yielded a median number of 18 QAPs (IQR 15-23). Whereas questions consisted of a median of 6 words (IQR 4-9), the answers consisted of a median of 22 words (IQR 15-29).

In our analysis of the participants’ wordings of questions, most questions did not show any abnormality (1673/1894, 88.3%). Foreign terms were found in 6.3% of the questions (119/1894), chain questions in 3.3% (n=62/1894), single-word questions in 1.2% (23/1894), and incomprehensible wording in 0.7% (13/1894). Four questions (0.2%) contained both a chain question and foreign terms.

### Quality of GPT-4’s Role-Play

To further assess GPT-4’s accuracy in providing a simulated patient chatbot, we assessed the quality of the role-play in the first 20% of conversations, which resulted in the analysis of 410 QAPs, as previously described [13].

Our script covered the majority of questions asked by participants (354/410, 86.3%), with 28 questions (6.8%) partly covered and 13 questions (3.2%) not covered at all by the script (not applicable: 15/410, 3.7%—that is, when no question was asked).

As for the answers provided by GPT-4, 99.3% of them matched the question (n=407), and no answer failed to match the question altogether (not applicable: n=3, 0.7%—that is, provided an answer to a previous question).

Regarding the plausibility of the answers provided by GPT-4, 99.3% (n=407) were rated as plausible, none as implausible, and 0.7% (3/410) as neither implausible nor plausible.

### Assessment of History Taking

#### Coverage of Feedback Categories and Items

**Figure 2:**
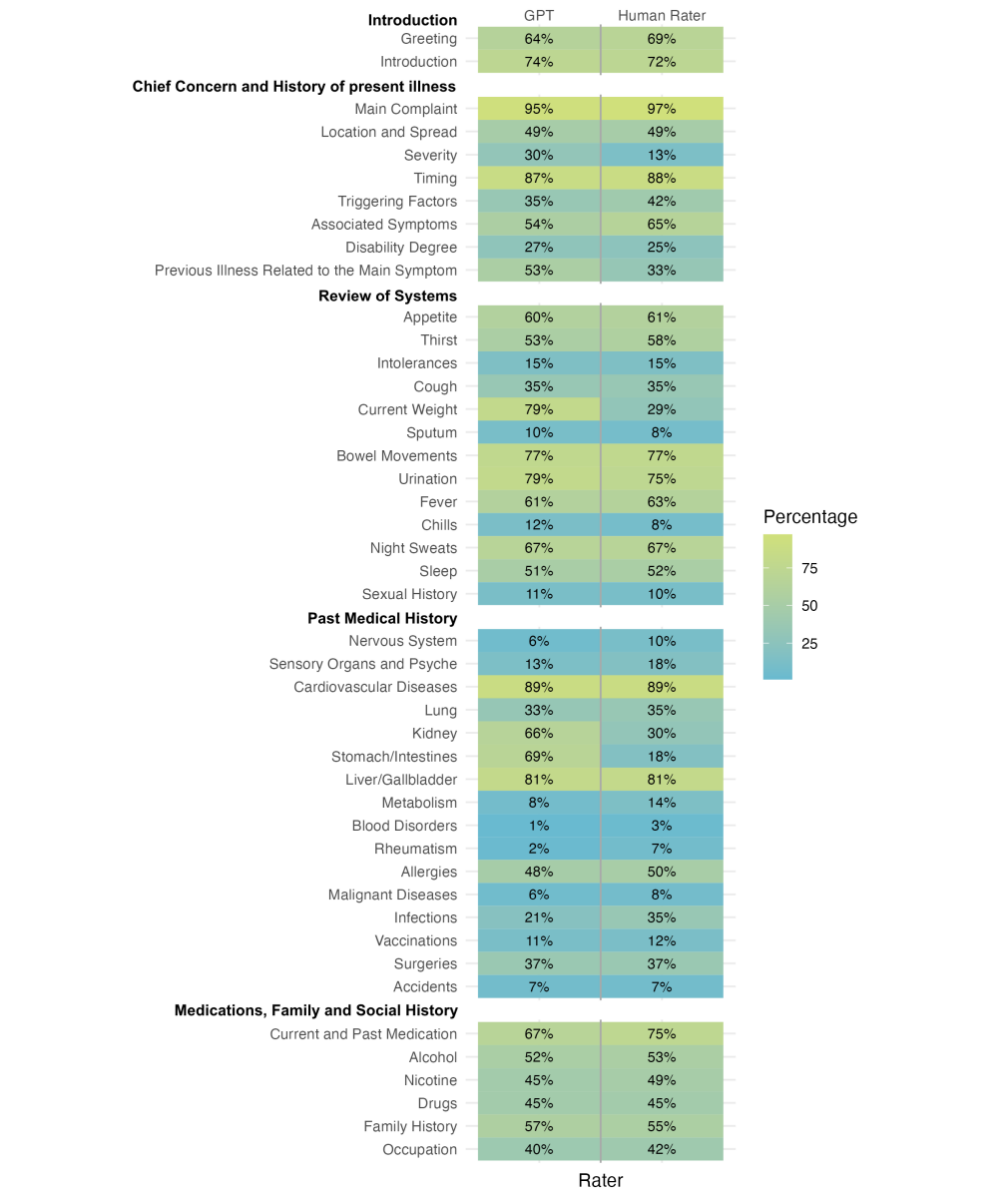
Heat map showing the percentage of conversations mentioning the feedback categories for both raters: Generative Pretrained Transformer (GPT) in the first column, and human rater in the second.

Participants’ history taking was assessed by both GPT-4 and the human rater (Figure 2). Combining both raters, the first feedback category (ie, introduction) was mentioned by 69.6% of participants, whereas the second category (ie, main complaint) was addressed by 52.7%. A total of 45.1% of participants asked about the vegetative system, and a system assessment was performed by 29.7% of participants. The fifth feedback category (ie, medication, family, social environment, and drugs) was addressed by 52% of participants.

#### Interrater Reliability

**Figure 3:**
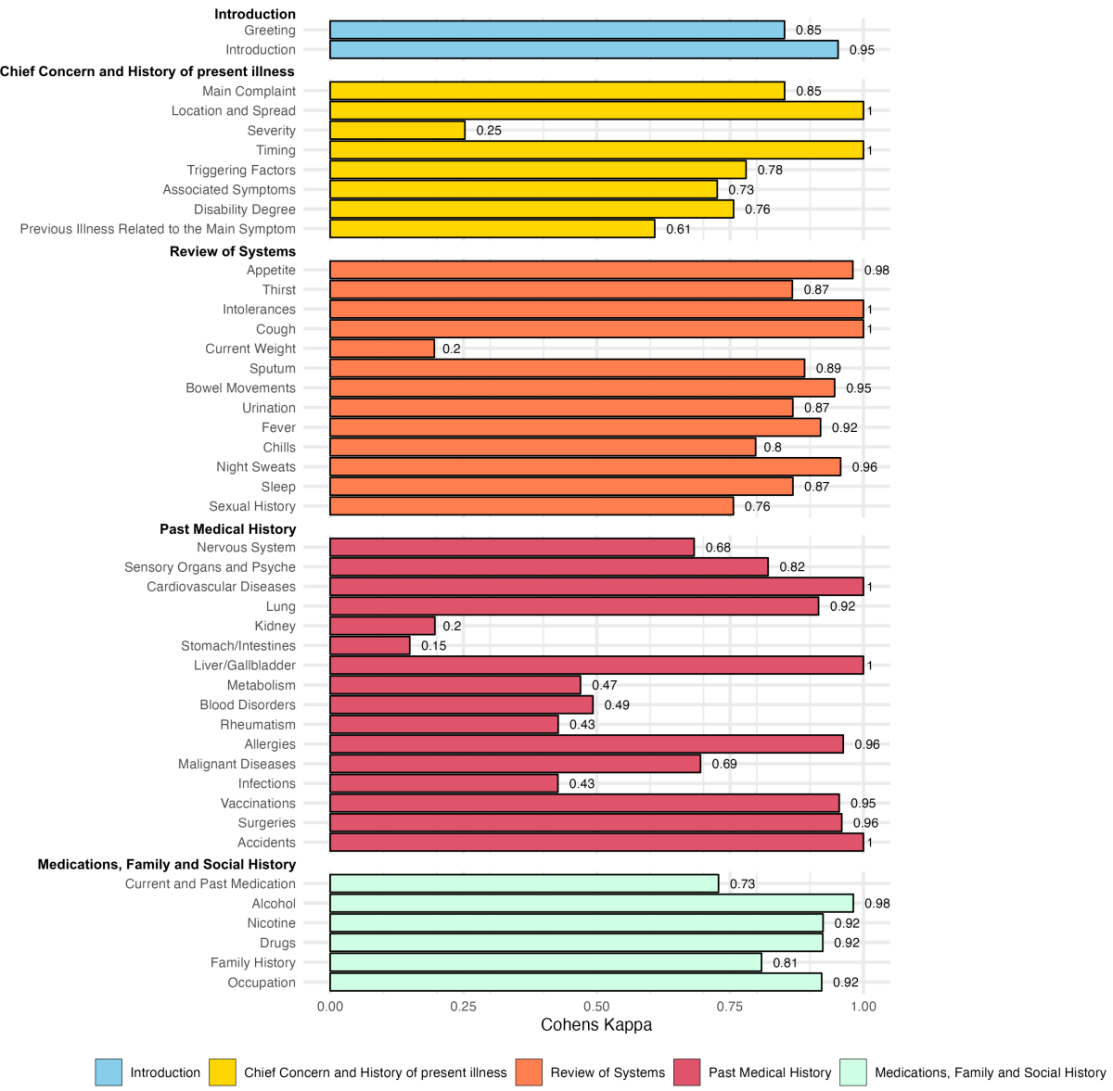
Cohen κ for every category of feedback for the human rater and Generative Pretrained Transformer (GPT) as a rater, with the different feedback topics displayed in different colors.

For total feedback, we found an interrater reliability, measured by Cohen κ, of 0.832 (95% CI 0.816-0.848), indicating an “almost perfect” agreement [32]. We further analyzed Cohen κ for each individual category of feedback, displayed in Figure 3.

#### Analysis of Divergent Ratings

As displayed in Figure 3, we found at least substantial interrater agreement for most categories of feedback. If conversations had divergent ratings, then we first inspected them in detail to evaluate whether agreement between the human rater and the GPT rating could be achieved. After corrections, 8 out of 42 categories still demonstrated lower-than-expected agreement (κ<0.6) and were, thus, further inspected (Table 2). For those categories, we performed a throughout analysis of the ratings and discussed possible reasons for the divergent ratings.

**Table 2:** List of feedback categories with Cohen κ<0.6.

Feedback category	Cohen κ	Mentioned (GPT)	Mentioned (Human rater)	Probable explanations for low Cohen’s κ with suggested solution and specific example (if appropriate)
Severity	0.25	30%	13%	The category “Severity” derived from a pain history. In the context of the illness script, there was overlap with the category “disability degree.” Suggested solution: Clarify category “Severity” and possibly rename it “Pain, Numeric Analogue Scale.” Specific example (from the illness script): Severity: “Recently, I’ve been significantly restricted. In the evenings after a long workday, I can’t do anything, and I’ve also noticed that I keep forgetting things at work.” Disability degree: “By now, I feel severely limited. This can’t continue. I can’t manage either my work or the tasks at home with my family like this!”
Current weight	0.20	79%	29%	Probably different interpretation: GPT was more liberal than the human rater. For example, when students asked any question related to weight, GPT rated it as “yes,” whereas the human rater rated it as yes only when actual weight was mentioned. Suggested solution: Define category more precisely or split category in “Current Weight” and “Weight Dynamics.” Specific example (from the illness script): “Overweight, previously 115 kg at a height of 178 cm, but now I only weigh 105 kg.”
Kidney	0.20	66%	30%	Polyuria has been repeated in the category “Kidney” because it was deemed highly important information. However, it resulted in an overlap with the category “Urination.” Suggested solution: Give information only once and precisely. Specific example (from the illness script): Urination: “Lately, I’ve been experiencing frequent urination during the day and at night. There’s no pain during urination, and the urine looks normal, as usual.” Kidney: “No pre-existing conditions, but now I constantly have to go to the toilet at night. However, I also haven’t been to a urologist in a long time.”
Stomach or intestines	0.15	69%	18%	Overlap exists with the category “Bowel Movements,” however, medically challenging to separate clearly. Suggested solution: Amend prompt to instruct GPT to rate both categories as “Yes” when a question or its answer covers both categories clearly and completely. Specific example (from the illness script): Stomach or intestines: “Mild tendency towards constipation” Bowel Movements: “Tending more towards constipation, but recently having a regular bowel movement once a day. Stool is otherwise normal: brown, without blood, without mucus, and without diarrhoea.”
Metabolism	0.47	8%	14%	Probably a different interpretation: GPT did not rate conversations positively when students asked for “metabolism disorders” and “diabetes.” Because we could not explain those ratings, we prompted GPT to explain its reasoning. The answer included that metabolism “encompasses all the chemical reactions that occur in the body” and includes aspects on “how [the] body converts food into energy,” thereby confirming our suspicion of different interpretations. Example of a question rated “Yes” by human rater and “No” by GPT: “Are you aware of having diabetes or hypercholesterolemia?”
Blood disorders	0.49	1%	3%	Low prevalence of “Yes” in the feedback category [33]
Rheumatism	0.43	2%	7%	Low prevalence of “Yes” in the feedback category [33]
Infections	0.43	21%	35%	Category not defined clearly enough with overlaps between “recent infections” and “infectious diseases.” Suggested solution: Amend illness script to include both categories and define both categories clearly. Example of statement rated “Yes” by GPT and “No” by human rater: “Additionally, I suffer from many simple infections, an increased sense of thirst, and dizziness.”

## Discussion

In our study, we assessed GPT-4’s performance in providing automatic feedback on learners’ history taking in a large cohort of medical students. Our findings suggest that GPT-4, accessed via an API, is capable of not only simulating patient experiences through a chatbot-like interface but also of providing accurate feedback on medical history-taking dialogs.

### Principal Results

Extending the line of our group’s previous research, the study presented here confirmed GPT-4’s capability of offering medically plausible responses in more than 99% of interactions, with a negligible rate of missing server responses (1.4%) that showcases its high reliability and availability in medical training [13]. That technical capability is particularly relevant when considering the asynchronous nature of such feedback systems in educational settings [34]. Building on our past work [13], we have demonstrated that GPT-4 can not only act as a simulated patient chat bot but can also assist the learner in providing structured feedback on the topics covered or not covered by the student.

The high level of agreement (Cohen κ=0.832) between GPT ratings and human ratings of students’ input that we observed indicates GPT-4’s capabilities in evaluating history-taking dialogs. It also supports GPT-4’s potential to enhance medical education by providing immediate, accurate feedback to students, thereby potentially fostering the learning process by enabling more practice opportunities and instant feedback. Given the importance of feedback for the learning process, the result offers an encouraging perspective on how LLMs such as GPT-4 can be used to cultivate the skills acquisition of medical students [17,18].

At the same time, we also found 8 feedback categories that yielded a Cohen κ of less than 0.6. For those items, in some cases we found GPT-4 to be “overly specific” in its rating. For example, in the category “Current Weight,” GPT-4 rated the occurrence of the topic “weight” positively (ie, disregarding whether the actual weight was mentioned), whereas the human rater focused on whether the actual weight was present in the chat. Those cases can probably be attributed to different interpretations of the items rated, and they indicate that the prompting should be as specific as possible in order to achieve higher interrater reliability.

We further hypothesize that those ratings can be improved by providing more detailed specifications for every category—for instance, by including examples and using more advanced prompting techniques such as chain-of-thought prompting [35]. However, longer prompts might be problematic when using models such as GPT-4, for the context window is limited to 8192 tokens [36]. Although our prompts (ie, system prompt of 2303 tokens and feedback prompt of 1336 tokens) fit well within those limits, longer prompts could require more advanced LLMs with longer context windows.

Furthermore, some lower κ values could have been caused by certain categories overlapping with other categories (eg, “Kidney” and “Urination”). Because medical cases often affect multiple topics, future studies should focus on the clear separation of feedback items. In our study, we did not prompt GPT-4 to provide any reasoning for the ratings (eg, in “chain-of-thought” prompting [37]), which researchers could improve upon in the future in order to better understand the models’ output.

Regarding the performance of the participating students, completeness scores for the feedback topics ranged from 31.0% to 68.9%. Although such rates might seem to indicate only modest performance, students also had a time restriction of 8 minutes maximum (ie, owing to the practising circuit that our chatbot was embedded in), which made a complete history-taking dialog exceptionally difficult.

### Comparison With Prior Work

Since the development of digital learning systems, automatic feedback has emerged as a topic of interest. Covering the pre-LLM era, a systematic review from 2021 analyzed 63 studies, most of them examining programming and mathematical skills [23]. While the review’s authors concluded that automatic feedback can foster students’ performance, the main method of generating automatic feedback was a comparison with a desired answer [23]. Further developments then included sophisticated dialog management systems [38], although those systems still performed below the level of feedback generated by LLMs. Because those pre-LLM technologies have been shown to help students [23], it can be expected that properly employed LLMs might provide even more benefits to learners (although the comparison was not investigated in our study).

Consequently, the recent emergence of LLMs such as GPT has been heralded as having the potential to revolutionize how students learn [39]. For example, Dai et al [40] found that ChatGPT was capable of generating more detailed feedback than human instructors while also achieving high agreement with the instructor. Beyond that, and in line with our results, in a study with students learning English as a new language, feedback from GPT-4 was found to be of similar quality to human feedback regarding learning outcomes and students’ perception [41]. Furthermore, LLM-based feedback has been shown to elucidate secondary effects, including increasing positive emotions and task motivation [42]. Indeed, the high motivation of students to participate in our study and in past investigations supports that motivational aspect [13]. Another essential aspect is the curricular implementation of the feedback, which is important for learners to develop a widespread understanding and develop mastery [10]. However, when implemented correctly, LLMs offer new tools for education and can be further improved when combined with speech-to-text tools and personalized databases [43].

However, some studies have also revealed problems with AI-generated feedback. For example, one showed that some participants might have negative attitudes toward the feedback due to being AI-generated feedback [44]. Such attitudes could affect learning outcomes considering that students’ perception of feedback is associated with self-regulated learning [45]. Furthermore, LLMs might elicit unexpected behaviors and escape prompts, thereby resulting in problematic interactions [46]. Although we did not observe that unexpected behavior in our study, the feedback provided by the AI might ultimately be understood as “official” feedback and should thus be rigorously assessed for its quality. Last, incorporating AI in teaching might lead students to rely on AI instead of learning from it [47], which indicates the importance of keeping the complete learning task in mind when designing AI-based learning opportunities.

### Limitations

Our findings have some limitations that deserve discussion. First, we relied on 1 LLM (ie, GPT-4) and a single prompt in our study. Although our study has demonstrated GPT-4’s potential in medical education, our reliance on a single LLM and type of prompt means that our findings might not apply to all educational contexts. Future research should, therefore, explore a variety of prompts and LLMs. Second, we chose a specific case for the history-taking dialog. Although we believe that GPT-4’s observed performance is transferable, our data cannot corroborate that assumption. Exploring a variety of cases and conditions would provide a more robust understanding of GPT-4’s applicability and limitations. Third, we used binary criteria (ie, “yes” or “no”) for the completeness of history taking in order to provide students with a simple checklist on what was asked or not asked. However, real-world clinical dialogs and history taking are complex and might benefit from more nuanced evaluation in order to accurately reflect which skills and topics students need to improve upon. Beyond that, it is important for students to receive feedback from the AI-generated tool on their social skills (eg, nonverbal communication and comprehensible language) during patient-physician encounters, which should be further investigated in future research. Last, we did not measure any educational outcomes (ie, skill acquisition), and thus, cannot state whether the AI-generated feedback in fact improved students’ performance.

### Conclusions

In sum, the LLM GPT-4 can provide a simulated patient experience and generate tailored, unsupervised feedback for medical students. The feedback given by GPT-4 was mostly accurate and had few minor flaws, most of which likely stemmed from our prompts. Our findings support the implementation of the system and the evaluation of its effectiveness in subsequent assessments.

## Appendix

Multimedia Appendix 1 Full prompt.

## Abbreviations

AI: artificial intelligence
API: application programming interface
GPT: Generative Pretrained Transformer
LLM: large language model
QAP: question-answer pair

## Data Availability

The data sets used and analyzed in our study are available from the corresponding author upon reasonable request.
